# Occupational therapists, self‐regulation, and primary schools: A scoping review

**DOI:** 10.1111/1440-1630.70101

**Published:** 2026-07-12

**Authors:** Lydia Cossart, Nikos Thomacos, Anoo Bhopti, Ellie Fossey

**Affiliations:** ^1^ Department of Occupational Therapy, School of Primary and Allied Health Care Monash University Frankston Victoria Australia; ^2^ Department of Psychology, School of Health and Biomedical Sciences RMIT University Melbourne Victoria Australia

**Keywords:** occupational therapists, occupational therapy, primary schools, self‐control, self‐regulation, students

## Abstract

**Introduction:**

The primary school setting is a dynamic practice context for occupational therapists. Despite a clear interest in understanding and intervening in self‐regulation in the classroom, little school‐specific literature is available.

**Methods:**

A scoping review was conducted to examine how occupational therapists understand self‐regulation in the mainstream primary school setting. The literature was searched as follows: occupational therapists (population [P]); self‐regulation intervention, assessment, or program (concept [C]); and education, school, and classrooms (context [C]).

**Consumer and Community Involvement:**

No consumers were directly involved in this review. The topic was informed by occupational therapy practice with teachers and families to ensure relevance to school and community needs.

**Results:**

Literature was scoped from five key health databases (CINAHL Complete, Embase via Ovid, ERIC, Ovid MEDLINE, and APA PsycInfo), with 192 papers initially identified. Two authors completed initial title and abstract screening, and full‐text papers were reviewed by the entire research team. Data were charted into tables following the Preferred Reporting Items for Systematic Reviews and Meta‐Analyses (PRISMA) guidelines and JBI Manual for Evidence Synthesis. Papers were included if occupational therapists were the main population of interest; included an intervention that improved classroom self‐regulation skills (student or teacher), and occurred in a mainstream primary school (public or private).

**Conclusion:**

Twelve papers were included in the final review. Seven papers were from a peer‐reviewed source, and five papers were dissertations (i.e. grey literature). Self‐regulation continues to be incompletely considered in the included papers, with papers either focussed on related constructs or on self‐regulation‐targeting programmes. Self‐regulation was also not well understood occupationally, with little to no operationalisation in terms of students' school‐related occupational performance and/or engagement outcomes. Further research is needed to appropriately conceptualise, define, and map self‐regulation occupationally.

## PLAIN LANGUAGE SUMMARY

Self‐regulation helps people have control over their thoughts, feelings, and behaviour. Primary school students require self‐regulation to follow directions and learn in the classroom setting. While occupational therapists work with primary school children to develop these skills, it is not yet clear how occupational therapists understand self‐regulation and what approaches and strategies they use consistently to develop self‐regulation in classroom settings. Some occupational therapists use structured programmes, whereas others use a variety of strategies to support primary school students. This scoping review focusses on what occupational therapists think about self‐regulation when working in primary schools. More research is needed to explain how self‐regulation is understood and what occupational therapists do when helping primary school students with self‐regulation concerns to build their skills.

**Key Points for Occupational Therapy**

- Occupational therapists' understanding of self‐regulation and related interventions is still emerging in the primary school context.
- Discussion of self‐regulation is common in occupational therapy literature, and occupational therapists commonly work towards supporting students' self‐regulation skills by using manualised and structured programmes.
- Self‐regulation is not well understood from an occupational perspective, and further research is needed to explore self‐regulation in the primary school environment

## 1 INTRODUCTION

Self‐regulation is a critical concept of interest across the lifespan (Geldhof et al., 2017; Rauvola & Rudolph, 2023; Vink et al., 2020). Many disciplines have researched self‐regulation, including psychology and education, leading to variability in how the concept has been conceptualised and operationalised. Broadly, self‐regulation is a multi‐dimensional term involving the management of emotions, attention, and behaviour (Blackwell et al., 2014). Self‐regulation is goal‐directed and is often closely linked with self‐control and self‐management (Grimmer, 2022; Groß, 2021; Hennecke & Burgler, 2022).

Early childhood is a critical period for the development of self‐regulation. For example, Montroy et al. (2016) and Wyatt et al. (2025) describe that from age 3 to 7 years, children shift from experiencing reactive behaviours that are managed by adults (otherwise termed co‐regulation) to predominantly cognitive‐driven behaviours (otherwise termed autonomy or self‐regulation). This shift occurs as children age, gain more life experiences, and thus have time to develop and learn from warm and trusting interactions and relationships with others (Grimmer & Geens, 2022). Self‐regulation skills can, therefore, be considered foundational skills for life and support improved life course outcomes (e.g. positive behavioural health trajectories and academic achievement) (Day et al., 2022; Miller et al., 2020; Solomon et al., 2018; Timmons et al., 2016). Aside from the benefits accruing to the child, it is important for society to support the successful and ongoing development of self‐regulation skills in young children, as those with highly developed self‐regulation skills are less likely to engage in risky behaviour, such as breaking the law or experiencing drug‐ and alcohol‐related disorders (Cooper et al., 2023; Howard & Williams, 2018; Robson et al., 2020). Specifically, the relevance of self‐regulation in these studies is suggested as supporting behaviour that resists or, in some cases, overrides impulsive and/or habitual behaviour commonly associated with risky behaviour, breaking the law, and/or drug and alcohol‐related disorders (Elhusseini et al., 2022). Considering the life course specifically, supporting the development of self‐regulation at a young age also benefits the individual over the life course, as responsive and adaptive self‐regulation skills have been associated with overall life success (e.g. school readiness, academic achievement, improved sense of self‐worth and developed adaptive coping skills) (Montroy et al., 2016; Zakszeski et al., 2020).

### 1.1 Self‐regulation, school, and occupational therapy

School is a critical environment for developing self‐regulation skills (Dettmer et al., 2020; Muir et al., 2023). Children experience a significant shift in attachment to others and behaviour when transitioning from the home to the school environment (Portilla et al., 2014). This sensitive period of development from approximately 5 to 12 years of age is characterised by a rapid acquisition of skills in many areas, including self‐regulation skills (Findik & Yildiz, 2014). The transition to primary school also represents a shift from home‐based relationships (i.e. child–parent/carer relationship) to a broader set of relationships (e.g. child–child and child–teacher relationships) (Hu et al., 2021; Moos & Ringdal, 2012; Portilla et al., 2014). Without explicit adult support during this critical transition period, some children will thrive, while others will not meet the demands of primary school classroom environments, and consequently, are more likely to have reduced school engagement, including poorer long‐term academic outcomes, as they transition into high school and beyond (Allan et al., 2014; Bosman et al., 2025; Heckhausen & Wrosch, 2016; Larsen et al., 2025; Varghese et al., 2019). Thus, teachers, as key adults in the primary school setting, are critical facilitators of childhood development (Rosanbalm & Murray, 2017). Research has found that sensitive and responsive primary school teachers can effectively buffer the effects of negative family environments (Portilla et al., 2014). Similarly, well‐developed self‐regulation skills have also been found to buffer the impacts of growing up in household chaos (Crespo et al., 2019). Consequently, developing self‐regulation skills in the primary school environment is increasingly becoming of interest to educators to manage classroom behaviour and ultimately improve educational and social outcomes (Blackwell et al., 2014; Lynch et al., 2023; McClelland et al., 2010; Muir et al., 2023; Torrington et al., 2023; Williams et al., 2023).

In a mainstream school setting, the role of occupational therapy is dynamic (Ball, 2018; Grajo et al., 2020; Laverdure, 2014; Smiles et al., 2025). Occupational therapy interventions in this environment are focussed on supporting primary school students to access and participate in all school activities, and occupational therapists in this role are well positioned to enable students to build the skills necessary for school (Christner, 2015). Since the origins of primary school‐based occupational therapy in the 1970s, the role and scope of practice has expanded beyond direct intervention (e.g. one‐on‐one direct therapy) to include programme delivery (e.g. the Alert Program® in Gill et al. [2018], McQuaid [2018], Novak and Honan [2019]) and school‐wide support (e.g. positive behaviour support in Hintz et al. [2022], Seruya and Garfinkel [2020], Smith and Douglas [2022]). Similar growth in the scope of practice has been seen in occupational therapists moving from working solely with primary school children with disabilities or additional needs to working with all primary school students and staff. Kennedy and Stewart (2012) describe that most primary school‐based occupational therapists work within diverse models of support that involve direct intervention with children, consultation with teachers, and collaboration with staff more generally.

Collaboration is identified in the literature as a critical component of occupational therapists' primary school‐based practices (Jeremy et al., 2025); however, Kennedy and Stewart (2012) found that teachers and therapists often report a desire for collaboration, but implementation is less common. They comment that this mismatch is largely because of various cultural, organisational, and personal barriers that need to be carefully examined and addressed through professional development for those working in primary schools. Collaboration among occupational therapists and teachers has been explored extensively in school‐based practice (e.g. Kennedy, 2011; Kennedy and Stewart, 2012; Vincent et al., 2008), and recently in works by Jill Jeremy and her colleagues (Jeremy et al., 2024; Jeremy et al., 2025). Jeremy and colleagues reported that three key factors impact effective collaboration among occupational therapists and teachers: individual factors (e.g. communication challenges and developing clear agreed channels, mutual respect, and professional trust), professional factors (e.g. understanding of respective roles and scope), and environmental factors (e.g. administrative support and adequate resourcing, particularly time resources) (Jeremy et al., 2024).

### 1.2 Occupational therapy and self‐regulation

Three seminal scoping reviews unpack the connection between self‐regulation and occupational therapy. These reviews were published in 2016, 2023, and 2024, and explore how occupational therapists define self‐regulation and measure the construct (Martini et al., 2016; Philpott‐Robinson et al., 2023; Philpott‐Robinson et al., 2024). All three reviews discussed self‐regulation in the context of primary schools, focussing on primary‐school aged children as a population group, but not where the intervention occurred (i.e. home, at school, in clinic, etc.). In their review, Martini et al. (2016) examined how occupational therapists define self‐regulation across the lifespan (infant, toddler, school‐age, adult, and senior), finding that only one third of the included papers provided an explicit definition of self‐regulation, and that despite a broad variability in settings, most papers were related to primary school‐aged children. The authors concluded that most papers focussing on a primary school setting cited sensory integration and sensory processing as self‐regulation's underlying theoretical framework (Martini et al., 2016). Martini et al. (2016) expanded on this conclusion, discussing how most included papers in their scoping review that utilised sensory frameworks rarely defined self‐regulation as a construct.

Philpott‐Robinson et al. (2023) built on the Martini et al. (2016) review by considering how self‐regulation is measured in preschool and primary school settings. The authors found that none of the included papers were published by or involved occupational therapists, concluding that occupational therapists do not have a consistent, occupationally grounded tool to measure self‐regulation in the early years of schooling (Philpott‐Robinson et al., 2023). They also concluded that the general lack of clarity and consistency in defining self‐regulation has contributed to the absence of empirical measurement of self‐regulation as a construct (Philpott‐Robinson et al., 2023). Philpott‐Robinson et al. (2024) further considered the conflicting definitions of self‐regulation in occupational therapy literature in a later paper, finding no clear consensus on the definition of the construct. Collectively, the findings from these three scoping reviews suggest that further exploration of how occupational therapists consider, understand, and apply the construct of self‐regulation is warranted.

### 1.3 The current study

Scoping reviews seek to answer broad research questions and clarify key concepts in the literature (Pollock et al., 2023), as well as to offer insight into subject areas that are emerging or poorly known (Peters et al., 2022). As little is understood about the role of occupational therapists in supporting the self‐regulation needs of students in primary school classrooms specifically, a scoping review methodology was identified as the most appropriate methodology to explore current occupational therapy approaches to defining self‐regulation in the mainstream primary school classroom environment. Thus, this scoping review aimed to build on the works by Martini et al. (2016), Philpott‐Robinson et al. (2023), and Philpott‐Robinson et al. (2024) by examining literature that considers occupational therapy, self‐regulation, and intervention within primary school settings. This review, therefore, only included papers that focussed on occupational therapy in the primary school environment, rather than papers that investigated specific disabilities or impairments or other environments. As such, this scoping review examined the following research question: how do occupational therapists understand self‐regulation in the primary school setting?

## 2 METHODOLOGY

This scoping review was conducted in accordance with the *JBI Manual for Evidence Synthesis* (Peters et al., 2020), which provided the methodological framework for defining the review question, identifying evidence, and synthesising findings. Reporting followed the *Preferred Reporting Items for Systematic Reviews and Meta‐Analyses – Extension for Scoping Reviews* (PRISMA‐ScR) checklist (Tricco et al., 2018) to ensure transparency and completeness. No protocol is available for this review. Literature was searched using the following PCC format: occupational therapists (population [P]); self‐regulation intervention, assessment, or programme (concept [C]); and education, school, and classrooms (context [C]).

### 2.1 Eligibility criteria

Papers that met the following criteria were included in this review:

1. Occupational therapists undertook all aspects of planning and delivery of the self‐regulation intervention.
2. The study included an intervention that specifically focussed on improving student or teacher classroom self‐regulation skills.
3. The intervention occurred in a primary school setting, including public and private schools.
4. The study was published in English.

The exclusion criteria for this scoping review were the following:

1. Involved other professionals conducting the self‐regulation intervention, for example, teachers, researchers, and so forth.
2. The intervention occurred outside of a primary school setting, for example, preschools, high schools, special schools, and other vocational settings.
3. The population of interest was disability specific, for example, Autism Spectrum Disorder or Developmental Coordination Disorder.

### 2.2 Search strategy

A comprehensive search strategy was developed for this review in consultation with an experienced research librarian. Preliminary results revealed a limited number of peer‐reviewed publications in this area. Thus, all types of literature were included in this review, including peer‐reviewed papers as well as grey literature, such as magazine articles and dissertations, to ensure a broad search and thus consideration of the topic. Search terms were also kept broad to include as much of the relevant literature as possible. Primary schools were selected as the setting of interest commensurate with the rapid acquisition of self‐regulation skills during this critical period. Special schools were considered out of scope for this review, as cognitive ability/disability was not considered aspects of self‐regulation in this review. As special school environments are specialised primary school educational settings, and students often present with multiple diagnoses, it was determined that the link between self‐regulation and cognitive ability or disability would better be explored in a separate scoping review.

Search terms included both mapped subject headings and key term searches. No geographical criteria were applied to the search, encouraging a diverse range of perspectives. The following electronic databases were selected for their relevance to the research topic: (a) CINAHL Complete, (b) Embase via Ovid, (c) ERIC, (d) Ovid MEDLINE, and (e) APA PsycInfo. All results were exported into the Covidence online platform (Veritas Health Innovation, 2024), with duplicates removed both by the Covidence programme (Veritas Health Innovation, 2024) and then manually by the first author. For feasibility, papers without an English translation were not considered for this review. Searching was also not restricted by publication date. Searching was completed in July 2024. Historical papers and all school settings were initially included (such as high school). As the term ‘primary school’ is not a universal term that refers to school‐aged children between 5 and 12 years of age, initial searching included all school settings (nursery, elementary, high school, etc.). Identified papers were then refined by hand to include only papers that focussed on the target population (i.e. children in primary school). Importantly, the term ‘self‐control’ was added as a key search term, as self‐control is inherently linked to self‐regulation (Fitzpatrick et al., 2014; Muir et al., 2023) and frequently appeared in database searches as the umbrella term under which self‐regulation appears. No other related term was selected, as the authors felt that other related constructs, such as self‐management, were too broad, as these constructs include unrelated factors such as nutrition, socio‐economic background, and language. Preliminary papers were also citation searched to identify any further relevant papers using *Google Scholar* and the *Monash University Library Database*. Full search strategy for CINAHL Complete is available as Supporting Information.

A tabular analysis was undertaken to systematically organise and examine the characteristics, methods, and key findings of the included studies. All data were recorded in a shared Microsoft Excel document. This document was reviewed by the researchers for similarities and connections between data points. Most data points were charted using tabular analysis. However, terms related to self‐regulation (Table 2) were analysed using numeral frequency and charted according to the frequency that each term appeared in the introduction section of each paper. This approach was chosen pragmatically, as the introduction section of the paper usually positions terms for the reader and explains their relevance in relation to other known phenomena. This method of collection was not calibrated against a known standard.

Self‐regulation interventions were charted according to their perceived relation to the *International Classification of Functioning (ICF)* (World Health Organization, 2002 *)* areas of body structures and function, or activities and participation. Interventions were assigned a broad category that reflects the overall purpose of the intervention and associated measurement. This process was collaborative and completed by the entire research team. The ICF was selected as a tool for data charting in order to purposefully consider participation, a key occupational consideration when unpacking self‐regulation. The final information extracted for the current review included citation details, population information, and the overall study design for each peer‐reviewed paper. Further information related to each included paper's research questions was gathered, such as how each paper defined self‐regulation, what intervention was undertaken, and what measurement tools were used to evaluate outcomes. Given that the included dissertations represent a body of work rather than a specific study, less information could be extracted that aligned with the inclusion criteria for the current review. Specifically, included dissertations were abstracted to include citation details, self‐regulation definition, associated intervention undertaken, and the overall theory that each dissertation considered. All peer‐reviewed papers were critically appraised using the Crowe Critical Appraisal Tool (Crowe, 2013) to examine the trustworthiness of the paper.

### 2.3 Positionality statement

Three of the authors are occupational therapists, and one is a psychologist. Two authors (first and third) currently work in paediatrics, supporting children, their families, and school teams to develop their self‐regulation skills. All authors are affiliated with a university and have postgraduate research experience, with the first author currently working towards a Doctor of Philosophy. This scoping review forms part of the first author's doctoral research. All authors are committed to client‐centred enablement and contributing meaningfully to improving the lives of Australian children.

## 3 RESULTS

### 3.1 Selection of sources of evidence

One hundred and ninety‐two papers were initially identified via database searching, with a further four papers identified by hand searching reference lists. Fifty‐six duplicates were removed, leaving 140 papers to be assessed for eligibility. Initial title and abstract screening were conducted by the first and last authors, with 33 papers remaining for full‐text screening by the entire research team. When authors disagreed, all authors met to discuss the full‐text paper given the review's inclusion and exclusion criteria. Discussion continued until authors reached a consensus.

**FIGURE 1:**
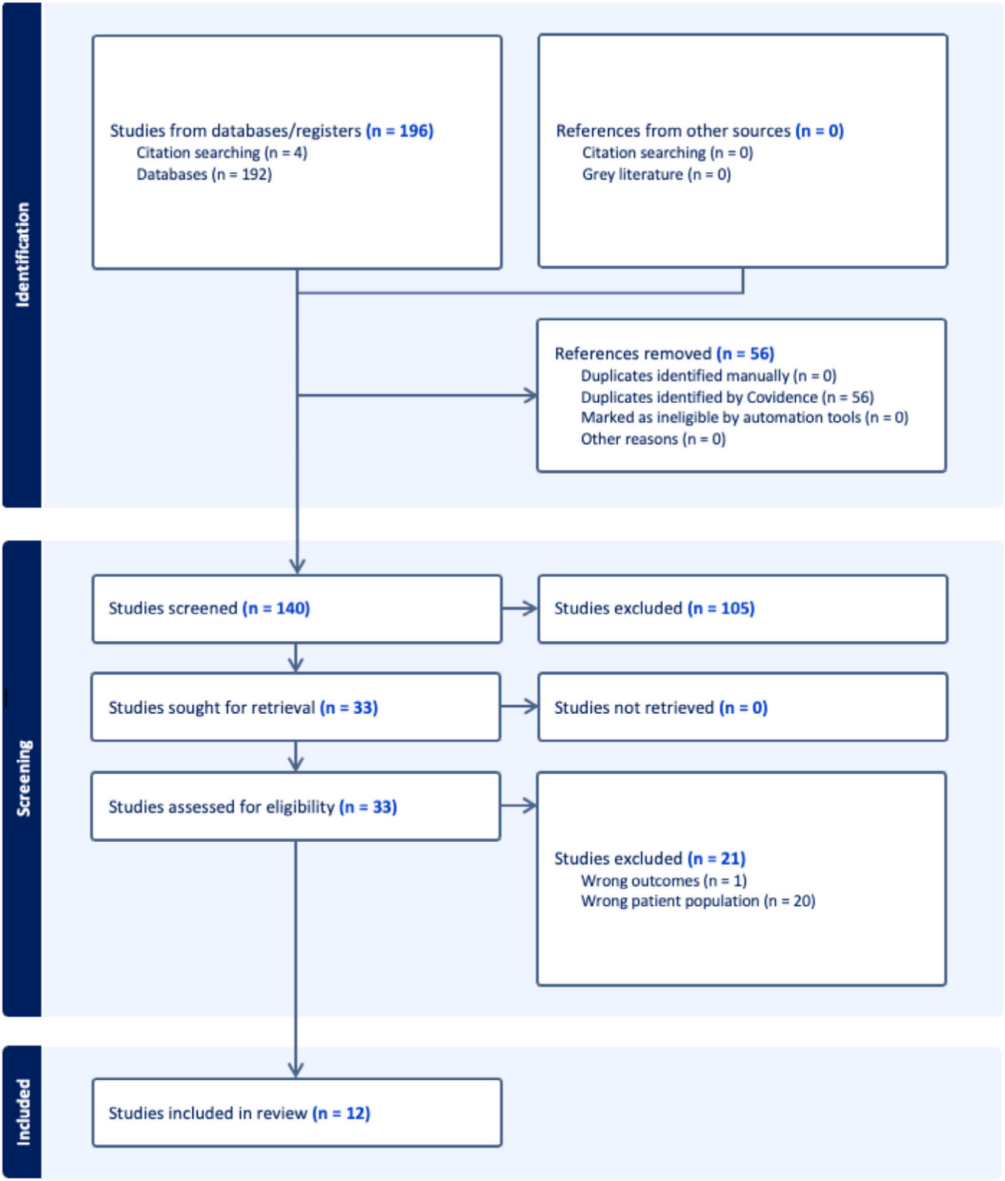
PRISMA flow diagram for the current scoping review. Note. Twenty‐six articles were excluded during the full‐text phase, primarily because they focussed on the wrong population (*n* = 20), such as high school or university students, or on the wrong outcome (*n* = 1), as the article measured hearing loss and not self‐regulation. PRISMA, Preferred Reporting Items for Systematic Reviews and Meta‐Analyses.

Twenty‐six papers were excluded during the full‐text screening phase, mostly as the papers focussed on the wrong population (*n* = 20), such as high school or university students, or the wrong outcome (*n* = 1) (i.e. the paper measured hearing loss and not self‐regulation). Papers focussing exclusively on a single aspect or dimension of self‐regulation, for example, social skills or socio‐emotional learning, were excluded as these papers were considered too narrow in scope and did not meaningfully consider self‐regulation as a holistic construct. Seven peer‐reviewed papers were found to meet the eligibility criteria, as did five grey literature publications (See the PRISMA Flow Diagram for a diagrammatic breakdown; Figure 1).

Results included peer‐reviewed journal articles and dissertations (i.e. grey literature). All papers and dissertations were authored by an occupational therapist. Included papers were published between 2016 and 2022, with authors from Australia, Canada, the USA, Spain, and Belgium. Included dissertations were published between 2015 and 2022. All peer‐reviewed papers were critically appraised and determined to be high quality, with the lowest *Crowe Critical Appraisal* score (Crowe, 2013) being 43 out of 50.

### 3.2 Data charting

Data were charted in a table, in line with the extraction protocol outlined in the *JBI Manual for Evidence Synthesis* (Peters et al., 2020). Data charting was initially completed separately by the first and last authors, with the factors chartered later reviewed and agreed upon by all research team members. Regular team debriefing and an audit trail were also established to increase the trustworthiness of the data charting process.

### 3.3 Synthesis of results

Studies were grouped by findings related to the research question, including how the authors defined self‐regulation and the intervention(s) and outcome(s) measured by each study. Results were abstracted and are presented in table format in Section 3 (Tables 1, 2, 3). Peer‐reviewed and grey literature were grouped separately, with Tables 1 and 2 presenting all information pertaining to the peer‐reviewed literature, and Table 3 presenting all grey literature information. Two key areas of interest emerged from the data charting—how self‐regulation was defined, and how self‐regulation was measured by occupational therapists in primary school settings.

**TABLE 1:** Peer‐reviewed papers with citation details, participant information and overall study design.

	Citation details			Participants		Study design			
	Author/s	Country	Aim/research question	Population	Sample size	Study design	Data collection method	Data analysis method	Crowe critical
**1**	Lin et al. (2022)	USA	To evaluate the effect of a universal mental health promotion programme on elementary school students in an underserved U.S./Mexico border community	Students in grade 5 and 6	85	Pilot study Quasi‐experimental design	Questionnaires	Descriptively via univariate analysis Paired sample *t*‐test	**49/50**
**2**	Romero‐Ayuso et al. (2022)	Spain	To analyse the effect of a self‐regulation programme at a primary school on the social interactions of children with special educational needs and neurotypical children from the teachers' and parents' perspectives	Primary school children (7–10 years of age)	107	Pilot study Pre‐test and post‐test design	Questionnaire	Kolmogorov–Smirnov test Chi‐square test Wilcoxon signed‐rank test	**49/50**
**3**	Wagner et al. (2020)	Australia	To assess the efficacy of an 8‐week Alert Program 1 intervention	Primary school students in remote first nations community (5.5–12.5 years of age)	271	Self‐controlled cluster randomised trial	Standardised assessment tools	Descriptive statistics	**46/50**
**4**	Challita et al. (2019)	Australia	To explore social skills of young children with reduced social incompetence	Children in kindergarten to grade 3	241	Descriptive, quantitative design using secondary data	Questionnaire	Descriptive analysis (paired sample *t*‐tests) Exploratory factor analysis	**48/50**
**5**	Gill et al. (2018)	Canada	To evaluate effectiveness, feasibility, and appropriateness of the Alert Program®	3 to 13 years	288	Systematic review	N/A	McMaster critical review criteria	**48/50**
**6**	Van Waelvelde et al. (2017)	Belgium	To investigate the effectiveness of the “I can!” program	Primary school children (7–8 years of age)	31	Pre–post design, including cross‐over	Standardised assessment tools	Shapiro–Wilk test Chi‐squared test Single and paired *t*‐tests	**44/50**
**7**	Hui et al. (2016)	Canada	To explore elementary school teachers perceived performance and satisfaction with their utilisation of learned strategies for students with disruptive behaviours and teacher self‐efficacy	Elementary school teachers	11	Pilot study. Multiple‐case replication design (semi‐structured interviews + intervention + pre–post data collection).	Standardised assessment tools	Person‐based analysis	**43/50**

*Note*: Papers displayed in order of newest to oldest publication date, showing the most contemporary considerations of self‐regulation concept first.

**TABLE 2:** Peer‐reviewed papers with aspects of self‐regulation considered, definition of self‐regulation, intervention considered, and dimension of occupational therapy measured.

	Citation	Self‐regulation definition			Self‐regulation intervention			
	Author/s	Self‐regulation directly defined	Self‐regulation (or related construct) definition	Related terms mentioned in introduction (number of times mentioned in introduction)	Programme applied	Measurement tool	Dimensions of occupational therapy measured (ICF)	Measured by
**1**	Lin et al. (2022)	No – Emotional self‐efficacy	‘One's perceived capabilities to recognise, comprehend and manage emotions to achieve a desirable outcome’	Social–emotional skills (2) Emotional self‐efficacy (8) Emotional regulation (1) Behavioural regulation (1) Emotional intelligence (1)	Zones of Regulation®	Self‐efficacy questionnaire for children (emotional domain)	Activities and participation	Students
**2**	Romero‐Ayuso et al. (2022)	Yes	‘The ability to control and modulate emotional expressions and to interact with others in an increasingly complex way according to social rules, adapt to emotionally challenging situations, and inhibit inappropriate behaviours’	Social–emotional skills/competence (7) Social and emotional learning (3) Social and emotional wellbeing (2) Self‐awareness (1) Social awareness (1) Executive functions (1) Effortful control (1)	The ‘exciting school’ programme	Peer social maturity scale (PSMAT)	Body structures and function	Teachers and parents
**3**	Wagner et al. (2020)	Yes	‘Volitional ability of a person to modify or maintain arousal states, emotions, thoughts, or behaviours appropriate to a situation’	Executive functioning (15) Sensory processing (1) Disruptive behaviour (4) Arousal states (2)	Alert program®	Sutter–Eyberg student behaviour inventory‐revised (SESBI) Eyberg child behaviour inventory (ECBI) Behaviour rating inventory of executive function 2 (BRIEF2‐SF)	Body structures and function	Teachers and parents
**4**	Challita et al. (2019)	No – Social competence	‘A broad multi‐ dimensional term used to describe how well children are able to evaluate social situations, and to select and apply social behaviours that are most appropriate to a given social context’	Social emotional learning (3) Social and emotional competence (1) Emotional competence (1) Emotional development (1) Social competence (10)	Nil programme applied	Questionnaire based on the perceive, recall, plan, and perform (PRPP) system of task analysis	Body structures and function	Parent and teacher
**5**	Gill et al. (2018)	Yes	‘The management of physiological arousal, emotions, attention, and behaviour’	Arousal (1) Emotion (1) Attention (1) Behaviour/socially approved behaviour (2) Social–emotional development (1) Self‐control (1)	Alert program®	NEPSY‐II (inhibition subtest) test of everyday attention for children (TEA‐Ch) Cambridge neuropsychological test automated battery (CANTAB) The behaviour rating inventory of executive functioning (BRIEF) (emotional control subscale) Wechsler abbreviated scale of intelligence and full scale BRIEF (emotional control subscale) MRI (structural of T1) The Roberts apperception test for children (RATC) Ready CLASS project activity assessment and school form Devereux early childhood assessment The sensory profile Self‐efficacy for self‐regulation of school‐aged children Teacher's perception of student's efficacy	Body structures and function Activities and participation	Teacher and student
**6**	Van Waelvelde et al. (2017)	Yes – self‐regulated learning	‘An active process whereby learners set learning goals and then attempt to monitor, regulate, and control their cognition, motivation, and behaviour, guided, and constrained by these goals and contextual features’	Self‐regulated learning strategy (3) Motivation (1) Behavioural problems (1)	I Can! Programme	Systematic screening of handwriting difficulties (SOS) Developmental test of visual‐motor integration 6th ed (VMI) Movement assessment battery for children 2nd ed (M‐ABC‐2 NL) One minute test (OMT)	Body structures and function	Therapists
**7**	Hui et al. (2016)	Yes	‘The ability to attain, maintain, and change the state of one's arousal level to attend to the task at hand’	Disruptive/challenging behaviour (3) Executive functions (1)	Alert Program® occupational performance coaching (OPC)	Canadian occupational performance measure (COPM) Goal attainment scaling (GAS) Teachers' sense of efficacy scale: Classroom management factor (TSES‐CM)	Activities and participation	Teachers

*Note*: Papers displayed in order of newest to oldest publication date, showing the most contemporary considerations of self‐regulation concept first.

**TABLE 3:** Grey literature with citation details, self‐regulation definition, intervention applied, and overall theoretical consideration.

	Paper type	Author/s	Year	Title	Self‐regulation definition	Intervention	Theory
**1**	Dissertation	Dash, M.	2021	Case study addressing disruptive behaviours of students with self‐regulation difficulties in the classroom	‘Being able to respond, process, and organise information adequately to adapt to the environment’	Nil applied	Ayres' sensory integration theory
**2**	Dissertation	Teselle, A.	2021	Linking body cues to emotions for elementary aged children: An understanding by design curriculum for social–emotional learning	‘Broad array of physiological, attentional, emotional, behavioural, cognitive, interpersonal and social processes’	My body feelings	Sensory processing Executive function
**3**	Dissertation	Costa, R.	2019	A classroom self‐regulation toolbox	‘Process of controlling one's emotions, behaviours, and thoughts, which supports adaptability for completing daily routines’	Classroom self‐regulation toolbox	Ayres' sensory integration theory Bandura self‐efficacy theory
**4**	Dissertation	Bateman, K.	2018	Moving to learn: Improving attention in the classroom setting for elementary school children	‘Methods used by students to manage, monitor, record, and/or assess their participation, work completion and behaviour and make meaningful improvements in on‐task behaviour, academic productivity and accuracy and reduction of inappropriate or disruptive behaviours’	Moving to learn	Sensory integration
**5**	Dissertation	Blackwell, A.	2015	The ready CLASS project: An examination of a tier 1 intervention in the early childhood classroom	‘Management of physiological arousal, emotions, attention, and behaviour’	The ready CLASS project	Sensory processing

*Note*: Papers displayed in order of newest to oldest publication date, showing the most contemporary considerations of self‐regulation concept first.

#### 3.3.1 Defining self‐regulation

Four of the seven peer‐reviewed papers and all dissertations included a definition of self‐regulation. All papers that directly defined self‐regulation (peer‐reviewed and grey) referenced varying definitions of self‐regulation (Tables 2 and 3). Instead of directly defining self‐regulation, one peer‐reviewed paper defined the related term ‘self‐regulated learning’, another paper defined ‘emotional self‐efficacy’, and a third paper defined the term ‘social competence’. Overall, self‐regulation was described as exerting control over one's own thoughts, feelings, and behaviours. Definitions also referenced appropriateness, which involves acting in accordance with social rules, being appropriate to a situation, or modulating oneself to attend to a task.

In the peer‐reviewed literature, self‐regulation was frequently defined alongside other similar key terms and constructs. In reviewing the papers (Challita et al., 2019; Gill et al., 2018; Lin et al., 2022; Romero‐Ayuso et al., 2022), the term ‘social emotional learning’ (or related terms ‘social emotional skills’, ‘social emotional competence’, ‘social emotional development’, or ‘social emotional well‐being’) was referenced most frequently, with the term mentioned 19 times in the introduction of the seven peer‐reviewed papers. ‘Executive function’ was the second most related term, with the term referenced 17 times in the introductions of the peer‐reviewed papers (Hui et al., 2016; Romero‐Ayuso et al., 2022; Wagner et al., 2020). The third most related term was ‘behavioural regulation’ (or related terms ‘disruptive behaviour’, or ‘socially approved behaviour’) tallied 11 times (Gill et al., 2018; Hui et al., 2016; Van Waelvelde et al., 2017; Wagner et al., 2020). The term ‘sensory processing’ was mentioned once in the introductory chapters of all seven included peer‐reviewed papers (Wagner et al., 2020).

#### 3.3.2 Measuring self‐regulation

Self‐regulation was not directly measured by any of the included peer‐reviewed papers (Table 2). Instead, authors used a variety of tools to measure performance outcomes, including a range of behaviour tools (such as the Behaviour Rating Inventory of Executive Functioning (BRIEF) (ACER, 2024) and the Devereux Behaviour Rating Scale (DBRS) School Form (Pearson Clinical, 2024), as well as goal‐focussed tools (such as the Canadian Occupational Performance Measure [COPM; The Canadian Occupational Performance Measure, 2024] and Goal Attainment Scaling [GAS; Assess Child, 2024]). These tools are all manualised, structured measurement tools. Similarly, none of the included peer‐reviewed papers directly mention self‐regulation in the research aim of the study. Four of the papers evaluated a programme, with two papers evaluating the effectiveness of an Alert Program® intervention, and the two other papers examining purpose‐designed programmes (‘I Can’ and the ‘Exciting School’ programmes).

Self‐regulation was measured using a variety of tools in the included peer‐reviewed papers (Table 2). Using the ICF (World Health Organization, 2002) to categorise the tools used, all measures related to either structures and function or activities and participation. Most measures related to body structure and functions. Of the included papers, three related to activities and participation (Gill et al., 2018; Hui et al., 2016; Lin et al., 2022). The tools focussing on domains of activities and participation were the Self‐Efficacy Questionnaire for Children (emotional domain), Self‐Efficacy for Self‐Regulation of School‐Aged Children, Teachers' Perception of Student's Efficacy, COPM, GAS, and Teachers' Sense of Self‐Efficacy Scale (Classroom Management Factor).

## 4 DISCUSSION

This scoping review aimed to expand occupational therapists' understanding of self‐regulation in a primary school classroom context. Seven peer‐reviewed papers and five dissertations were found to match the inclusion criteria of this review. The relatively small number of papers (particularly peer‐reviewed papers) suggests that research in this area remains a work in progress, particularly in the primary school setting. The grey literature further supports this conclusion, with the included dissertations resulting in a limited number of publications. Of the five dissertations included in this scoping review, only one author published beyond their thesis, with Blackwell (2015) publishing four times post their PhD. Three of these papers focussed on classroom interventions, and one on the post‐surgery needs of children. The scarcity of publications may highlight the need for innovative and rigorous methodologies and interdisciplinary collaboration to better capture how occupational therapists support the self‐regulation needs of primary school students.

This review affirms previous scoping reviews (Martini et al., 2016; Philpott‐Robinson et al., 2023), in that self‐regulation represents an ambiguous concept for occupational therapists. None of the included papers for this study were found in the included papers of the previous scoping reviews. Thus, new papers continue to affirm previous findings. In the current study, self‐regulation was seen to represent an active process, which involves control and modulation to achieve a specific goal. This definition may be reflective of the dominance and origins of self‐regulation as a psychological term (Billore et al., 2023). Defined psychologically by McCoy (2019, p. 64), self‐regulation can be considered through such a lens as it represents a ‘broad set of both conscious and unconscious processes that individuals use to regulate (e.g. control, modulate, inhibit, initiate) both their internal states (e.g. attention, emotion) and observable behaviour’. While this definition aligns with those in the papers included in the current review, such a perspective lacks a distinct occupational lens and does not consider domains of occupational performance, occupational participation, or occupational engagement.

Self‐regulation was not the major focus of most papers in this review, with self‐regulation rarely included in the research questions of each paper. Instead, included papers focussed on discrete aspects of self‐regulation, such as executive functioning and social–emotional skills, or on a programme such as the Alert Program® or Zones of Regulation® programme. This finding also reflects that no occupationally grounded self‐regulation tools are used in practice, aligning with the Philpott‐Robinson et al. (2023) review, that concluded similarly. Thus, if self‐regulation is not meaningfully operationalised and measured in terms of occupational performance or engagement outcomes, then it remains a construct that is closely aligned with other similar and measurable constructs (such as social–emotional learning and executive functioning).

In the included papers and dissertations, social–emotional learning was closely related to self‐regulation. This is reflected in broader literature (Rademacher & Koglin, 2019), with the two terms frequently appearing in tandem in literature (Bierman et al., 2008; Graziano & Hart, 2016; Liew, 2012). This can be understood by reference to Silkenbeumer et al.’s (2024) research that described how teachers play a key role in supporting the self‐regulation development of their students in the classroom. Specifically, teachers provide support in labelling emotions, validating emotional expression, and structuring the metacognitive process of regulating emotions (Silkenbeumer et al., 2024). Denham et al. (2012) relate self‐regulation and social–emotional learning skills as well as other related constructs, such as regulating attention and behaviour, as skills that ‘all work together to grease the cogs of a successful school experience’ (Denham et al., 2012, p. 178). Accordingly, self‐regulation and social–emotional skills can be considered distinct but closely related as constructs, with the development of social–emotional skills directly contributing to the development of self‐regulation.

Executive functioning frequently appeared in the peer‐reviewed and grey literatures. Cramm et al. (2013) suggest that terms, such as executive functioning, self‐awareness, self‐monitoring, and self‐regulation, are often used differently and/or in an ‘entangled’ manner in the occupational therapy literature. One explanation for this lack of consistency in terminology and/or application of terminology may be that these terms and constructs (i.e. executive functioning, self‐regulation, self‐control, effortful control) draw on similar underlying theories and at times shared processes, leading to definitional ambiguity (McClelland & Cameron, 2019). Executive functioning is inherently a cognitive construct and encompasses various skills and abilities such as such as working memory, shifting attention, and organisation of thoughts (Blair, 2017). Moreover, executive functioning skills have been referred to as the ‘crown jewel’ of self‐regulation, as they are believed to be the support mechanisms needed to achieve regulatory goals (Hofmann et al., 2012). The strong link proposed between executive functioning and self‐regulation in the literature helps to explain why the two terms often sit side‐by‐side in the occupational therapy literature, as self‐regulation can be considered to be the product of adaptive executive functioning. This may further contribute to their definitional ambiguity and occupational therapists' use of these terms relatively interchangeably.

Many included papers used a structured programme to target and support the development of self‐regulation skills in the primary school classroom. For example, Gill et al. (2018), Hui et al. (2016), and Wagner et al. (2020) utilised the Alert Program®; and Lin et al. (2022) used the Zones of Regulation® programme to support the development of self‐regulation in primary school‐aged children. These programmes incorporate multiple facets of self‐regulation and related constructs, such as executive functioning, sensory processing, and alertness to support children in developing the necessary skills to reach their goals.

In the included papers, the Alert Program® appears the most cited, with Hui et al. (2016) reporting that approximately half of all school‐based occupational therapists in the United States use the programme. The Alert Program® was developed by occupational therapists Williams and Shellenberger in 1996 and is based on Sensory Integration Theory (Mac Cobb et al., 2014). On the Alert Program® website, the programme's developers suggest that the programme explains sensory exposure and sensory diets to children in a novel format (using an engine analogy) and identifies five ways that children can influence their alertness: ‘put something in your mouth, move, touch, look, and listen’ (TherapyWorks, 2023). In this context, self‐regulation is described as ‘the ability to change how alert we feel’ (TherapyWorks, 2023). The Zones of Regulation® programme was also referenced in the included papers. Developed by occupational therapist Leah Kuypers in 2008, the programme aims to teach self‐regulation skills through participation in a structured, sequential programme (McQuaid, 2018). The Zones of Regulation® draws upon a range of theories and approaches, including Central Coherence Theory, cognitive behaviour management, trauma‐informed practice, and social–emotional learning frameworks (Mason et al., 2023; McQuaid, 2018). Despite the broad use of the programme, Mason et al. (2023) in their recent review concluded that limited empirical evidence exists regarding the outcomes of the Zones of Regulation® programme and that further studies evaluating the validity of the programme and its measurement tools are needed.

The connection between self‐regulation and sensory processing was explored in the delivery of both the Alert Program® and the Zones of Regulation® programme (Gill et al., 2018; Hui et al., 2016; Lin et al., 2022). Martini et al. (2016) and Philpott‐Robinson et al. (2023) similarly explored the link between the two concepts, finding that a sensory processing framework is used frequently by occupational therapists when considering the self‐regulation needs of their clients. Sensory integration is described as a neurological process focused on organising sensory information that enables participation (Andelin et al., 2021). The theory was conceptualised by Ayres in 1972 and later built upon by Winnie Dunn, with their formative Sensory Processing Framework defining self‐regulation as the management of sensory thresholds within the surrounding environment (Blackwell et al., 2014). Dunn further describes that self‐regulation occurs on a continuum whereby people respond passively or actively to their sensory environment (Dunn, 2011). Despite the broad acceptance of using a sensory integration lens when considering self‐regulation within an occupational context (as per Wagner et al. [2020] paper), occupational therapists are also moving towards a broader scope of intervention that encompasses multiple approaches to practice, as seen in programmes such as the Zones of Regulation®. Thus, reflecting the highly cognitive nature of self‐regulation, in the intervention itself.

Established programmes such as the Zones of Regulation® promote a cognitive approach to intervention, promoting a sense of agency as children learn the necessary skills to thrive in the classroom. Similarly, Occupational Performance Coaching, as seen in Hui et al. (2016), focusses on a complex and highly cognitive approach to intervention whereby clients (in this case, primary school students and teachers) are seen as active members of the intervention process, with agency and power. This approach lends itself to a tiered model of intervention, whereby primary school‐based occupational therapists provide one‐on‐one interventions alongside engagement in programmes and delivery of school‐wide support (Seruya & Garfinkel, 2020). In comparison, sensory integration and sensory processing are not as well aligned within a school‐wide approach to intervention, as therapists do not have the opportunity to provide specialist one‐on‐one assessment and intervention to address the needs of each primary school student. It is likely that occupational therapists working in primary schools and within a school‐wide model of support would therefore benefit from using a combination of approaches to self‐regulation intervention to support the best fit between the needs of the students and the specific primary school environment.

As identified in this scoping review, self‐regulation is inadequately understood from an occupational perspective in the primary school environment. Therefore, it is unclear how occupational therapists working in primary schools can best support students and their teachers. Continuing to look further afield to the psychology and education literatures, occupational therapists should build on these literatures to develop and build a comprehensive, top‐down, occupationally meaningful approach to intervention (by, for example, encompassing multiple approaches to practice that actively incorporate cognitive, social, and emotional domains). Such an approach, however, needs to ensure that self‐regulation is considered and operationalised from an occupational perspective in order to achieve a robust, evidence‐based occupational perspective and approach to self‐regulation.

### 4.1 Limitations

This study had several limitations. A limited number of papers were found to meet the criteria of this review. The study only included papers published in English, thus excluding potentially significant works published in other nations and languages. Papers were also excluded in this review if they focussed solely on a specific condition or neurotype, for example, Autism Spectrum Disorder. These papers were excluded from this review to align with the primary focus of understanding generalised school‐based supports provided by occupational therapists.

## 5 IMPLICATIONS FOR OCCUPATIONAL THERAPY PRACTICE

This scoping review, together with other recent seminal works (e.g. Martini et al., 2016; Philpott‐Robinson et al., 2023; Philpott‐Robinson et al., 2024), highlights the need for robust studies regarding self‐regulation and occupational therapy in the primary school setting. Future studies should consider:

1. establishing a deeper and more comprehensive understanding of why examining self‐regulation is important in occupational therapy practices in the primary school environment;
2. considering the current approaches to self‐regulation intervention by occupational therapists and how occupational therapists could measure the outcome(s) of their work in primary school classrooms; and
3. examining in greater detail how student occupational performance, engagement, and participation are influenced by occupational therapy self‐regulation interventions in the primary school context.

These studies would add significant depth and insight into the work of occupational therapists in support of the self‐regulation needs of their students at school.

## 6 CONCLUSION

Self‐regulation and occupational therapy are intrinsically linked as occupational therapists are increasingly considering the self‐regulation needs of their clients and that occupational therapists work to maximise their clients' performance and engagement. Such outcomes are especially critical in primary school‐aged children. Discussion of self‐regulation is common in the occupational therapy literature; however, there is ambiguity in how the construct is understood and defined in the primary school context, and self‐regulation is commonly not the direct focus of intervention. In the primary school setting, intervention in this area is mainly undertaken one‐on‐one by considering a related construct, such as executive functioning and sensory processing, or through the delivery of whole‐school programmes such as the Alert Program® and the Zones of Regulation®. Future research needs to consider self‐regulation from an occupational perspective, considering how the term can be understood using occupational models and theories, and how to support the self‐regulation knowledge and skills of students and their teachers in a primary school environment.

## AUTHOR CONTRIBUTIONS

Lydia Cossart completed this scoping review as part of a Doctor of Philosophy degree. Lydia was supervised during this review by Dr Nikos Thomacos, Dr Anoo Bhopti, and Professor Ellie Fossey. Lydia Cossart completed all data searching and analysis with ongoing guidance and review from the supervision team. All authors have contributed to the design of this scoping review and the drafting of the manuscript.

## CONFLICT OF INTEREST STATEMENT

The authors declare no conflicts of interest.

## Supporting information


**Data S1.** Supporting Information.

## Data Availability

The data that support the findings of this study are available from the corresponding author upon reasonable request.

## References

[aot70101-bib-0001] ACER . (2024). Behavior Rating Inventory of Executive Function – Second Edition (BRIEF2). https://shop.acer.org/behavior-rating-inventory-of-executive-function-second-edition-brief2.html

[aot70101-bib-0002] Allan, N. , Hume, L. , Allan, D. , Farrington, A. , & Lonigan, C. (2014). Relations between inhibitory control and the development of academic skills in preschool and kindergarten: A meta‐analysis. Developmental Psychology, 50(10), 2368–2379. 10.1037/a0037493 25069051

[aot70101-bib-0003] Andelin, L. , Reynolds, S. , & Schoen, S. (2021). Effectiveness of occupational therapy using a sensory integration approach: A multiple‐baseline design study. American Journal of Occupational Therapy, 75(6), 1–14. 10.5014/ajot.2021.044917 34817594

[aot70101-bib-0004] Assess Child . (2024). Goal Attainment Scale (GAS). https://assesschild.com/goal-attainment-scale

[aot70101-bib-0005] Ball, M. (2018). Revitalizing the OT role in school‐based practice: Promoting success for all students. Journal of Occupational Therapy, Schools & Early Intervention, 11(3), 263–272. 10.1080/19411243.2018.1445059

[aot70101-bib-0006] * Bateman, K. D. (2018). Moving to learn: Improving attention in the classroom setting for elementary school children (Publication No. 10683503) [Doctoral dissertation, Boston University]. ERIC. https://www.proquest.com/dissertations-theses/moving-learn-improving-attention-classroom/docview/2101590075/se-2?accountid=12528

[aot70101-bib-0007] Bierman, K. , Domitrovich, C. , Nix, R. , Gest, S. , Welsh, J. , Greenberg, M. , Blair, C. , Nelson, K. , & Gill, S. (2008). Promoting academic and social‐emotional school readiness: The head start REDI program. Child Development, 79(6), 1802–1817. 10.1111/j.1467-8624.2008.01227.x 19037951 PMC3549580

[aot70101-bib-0008] Billore, S. , Anisimova, T. , & Vrontis, D. (2023). Self‐regulation and goal‐directed behavior: A systematic literature review, public policy recommendations, and research agenda. Journal of Business Research, 156, 113435. 10.1016/j.jbusres.2022.113435

[aot70101-bib-0009] * Blackwell, A. (2015). The Ready CLASS Project: An examination of a Tier 1 intervention in the early childhood classroom: A pretest and posttest control group design (Publication No. 3719934) [Doctoral dissertation, University of Kansas]. ProQuest Dissertations & Theses. https://www.proquest.com/dissertations-theses/ready-class-project-examination-tier-1/docview/1718390489/se-2

[aot70101-bib-0010] Blackwell, A. , Yeager, D. , Mische‐Lawson, L. , Bird, R. , & Cook, D. (2014). Teaching children self‐regulation skills within the early childhood education environment: A feasibility study. Journal of Occupational Therapy, Schools & Early Intervention, 7(3–4), 204–224. 10.1080/19411243.2014.966013

[aot70101-bib-0011] Blair, C. (2017). Educating executive function. Wiley Interdisciplinary Reviews: Cognitive Science, 8(1–2), 1–6. 10.1002/wcs.1403 PMC5182118 27906522

[aot70101-bib-0012] Bosman, R. , de Jong, P. , & Koomen, H. (2025). Improving teacher‐child relationships using relationship‐focused reflection: A case study. Evaluation & the Health Professions, 48(1), 16–29. 10.1177/01632787241250366 39628359 PMC11909776

[aot70101-bib-0013] * Challita, J. , Chapparo, C. , & Hinitt, J. (2019). Patterns of social skill difficulties in young children with reduced social competence: Parent and teacher perceptions. Journal of Occupational Therapy, Schools & Early Intervention, 12(3), 298–310. 10.1080/19411243.2019.1590752

[aot70101-bib-0014] Christner, A. (2015). Promoting the role of occupational therapy in school‐based collaboration: Outcome project. Journal of Occupational Therapy, Schools & Early Intervention, 8(2), 136–148. 10.1080/19411243.2015.1038469

[aot70101-bib-0015] Cooper, D. , Felt, J. , Riobueno‐Naylor, A. , Lai, B. , Bámaca, M. , & Fishbein, D. (2023). The mediating role of self‐regulation on the link between child maltreatment and later substance use among Latinx youth. Child Abuse & Neglect, 140, 1–11. 10.1016/j.chiabu.2023.106151 PMC10164059 36965435

[aot70101-bib-0016] * Costa, R. (2019). A classroom self‐regulation toolbox: A collaborative program between occupational therapists and teachers for children with ADHD (Publication No. 22623482) [Doctoral dissertation, Boston University]. ProQuest Dissertations & Theses. https://www.proquest.com/dissertations-theses/classroom-self-regulation-toolbox-collaborative/docview/2318188199/se-2

[aot70101-bib-0017] Cramm, H. A. , Krupa, T. M. , Missiuna, C. A. , Lysaght, R. M. , & Parker, K. H. (2013). Executive functioning: A scoping review of the occupational therapy literature. Canadian Journal of Occupational Therapy, 80(3), 131–140. 10.1177/0008417413496060 24224226

[aot70101-bib-0018] Crespo, L. , Trentacosta, C. , Udo‐Inyang, I. , Northerner, L. , Chaudhry, K. , & Williams, A. (2019). Self‐regulation mitigates the association between household chaos and children's behavior problems. Journal of Applied Developmental Psychology, 60, 56–64. 10.1016/j.appdev.2018.10.005 31772417 PMC6879109

[aot70101-bib-0019] Crowe, M. (2013). Crowe Critical Appraisal Tool (CCAT). www.conchra.com.au

[aot70101-bib-0020] * Dash, M. (2021). Case study addressing disruptive behaviors of students with self‐regulation difficulties in the classroom (Publication No. 28860940) [Doctoral dissertation, Northcentral University]. ProQuest Dissertations & Theses. https://www.proquest.com/dissertations-theses/case-study-addressing-disruptive-behaviors/docview/2650272876/se-2

[aot70101-bib-0021] Day, N. , Paas, F. , Kervin, L. , & Howard, S. J. (2022). A systematic scoping review of pre‐school self‐regulation interventions from a self‐determination theory perspective. International Journal of Environmental Research and Public Health, 19(4), 2454. 10.3390/ijerph19042454 35206641 PMC8878745

[aot70101-bib-0022] Denham, S. A. , Bassett, H. , Mincic, M. , Kalb, S. , Way, E. , Wyatt, T. , & Segal, Y. (2012). Social–emotional learning profiles of preschoolers' early school success: A person‐centered approach. Learning and Individual Differences, 22(2), 178–189. 10.1016/j.lindif.2011.05.001 22408363 PMC3294380

[aot70101-bib-0023] Dettmer, A. , Clinton, A. , & Mildon, H. (2020). Self‐regulation in young children: A skill set for lifetime success. In Healthy development in young children: Evidence‐based interventions for early education (pp. 131–150). American Psychological Association. 10.1037/0000197-007

[aot70101-bib-0024] Dunn, W. (2011). Best practice occupational therapy for children and families in community settings (2nd ed.). SLACK. 10.3109/07380577.2012.717734 23899204

[aot70101-bib-0025] Elhusseini, S. A. , Tischner, C. M. , Aspiranti, K. B. , & Fedewa, A. L. (2022). A quantitative review of the effects of self‐regulation interventions on primary and secondary student academic achievement. Metacognition and Learning, 17(3), 1117–1139. 10.1007/s11409-022-09311-0

[aot70101-bib-0026] Findik, E. , & Yildiz, T. (2014). Preschool self‐regulation assessment (PSRA): Adaptation study for Turkey. Education and Science, 39(176), 317–328. 10.15390/EB.2014.3647

[aot70101-bib-0027] Fitzpatrick, C. , McKinnon, R. D. , Blair, C. B. , & Willoughby, M. T. (2014). Do preschool executive function skills explain the school readiness gap between advantaged and disadvantaged children? Learning and Instruction, 30, 25–31. 10.1016/j.learninstruc.2013.11.003

[aot70101-bib-0028] Geldhof, G. , Fenn, M. , & Finders, J. (2017). A self‐determination perspective on self‐regulation across the life span. In M. Wehmeyer , K. Shogren , T. Little , & S. Lopez (Eds.), Development of self‐determination through the life‐course (pp. 221–235). Springer Netherlands. 10.1007/978-94-024-1042-6_17

[aot70101-bib-0029] * Gill, K. , Thompson‐Hodgetts, S. , & Rasmussen, C. (2018). A critical review of research on the Alert Program®. Journal of Occupational Therapy, Schools & Early Intervention, 11(2), 212–228. 10.1080/19411243.2018.1432445

[aot70101-bib-0030] Grajo, L. C. , Candler, C. , & Sarafian, A. (2020). Interventions within the scope of occupational therapy to improve children's academic participation: A systematic review. The American Journal of Occupational Therapy, 74(2), 7402180030p7402180031–7402180030p7402180032. 10.5014/ajot.2020.039016 32204774

[aot70101-bib-0031] Graziano, P. A. , & Hart, K. (2016). Beyond behavior modification: Benefits of social–emotional/self‐regulation training for preschoolers with behavior problems. Journal of School Psychology, 58, 91–111. 10.1016/j.jsp.2016.07.004 27586072

[aot70101-bib-0032] Grimmer, T. (2022). Development of self‐regulation. In T. Grimmer & W. Geens (Eds.), Nurturing self‐regulation in early childhood: Adopting an ethos and approach (1st ed.) (pp. 24–44). Taylor & Francis Group. 10.4324/978100316234610.4324/9781003162346

[aot70101-bib-0033] Grimmer, T. , & Geens, W. (2022). Nurturing self‐regulation in early childhood: Adopting an ethos and approach. Taylor & Francis Group.

[aot70101-bib-0034] Groß, D. (2021). In the self‐control and self‐regulation maze: Integration and importance. Personality and Individual Differences,175, 110728. 10.1016/j.paid.2021.110728

[aot70101-bib-0035] Heckhausen, J. , & Wrosch, C. (2016). Challenges to developmental regulation across the life course: What are they and which individual differences matter? International Journal of Behavioral Development, 40(2), 145–150. 10.1177/0165025415588796

[aot70101-bib-0036] Hennecke, M. , & Burgler, S. (2022). Metacognition and self‐control: An integrative framework. Psychological Review, 130(5), 1–27. 10.1037/rev0000406 36521121

[aot70101-bib-0037] Hintz, L. A. , Fletcher, T. , Cahill, S. , & Poskey, G. (2022). Teachers' experiences with occupational therapy multi‐tiered systems support: A qualitative study. Journal of Occupational Therapy, Schools & Early Intervention, 16(4), 432–449. 10.1080/19411243.2022.2112361

[aot70101-bib-0038] Hofmann, W. , Schmeichel, B. J. , & Baddeley, A. D. (2012). Executive functions and self‐regulation. Trends in Cognitive Sciences, 16(3), 174–180. 10.1016/j.tics.2012.01.006 22336729

[aot70101-bib-0039] Howard, S. J. , & Williams, K. E. (2018). Early self‐regulation, early self‐regulatory change, and their longitudinal relations to adolescents' academic, health, and mental well‐being outcomes. Journal of Developmental & Behavioral Pediatrics, 39(6), 489–496. 10.1097/DBP.0000000000000578 29781830

[aot70101-bib-0040] Hu, B. Y. , Guo, Y. , Wang, S. , & Vitiello, V. E. (2021). The associations between teacher‐child relationships and academic skills: A longitudinal study among Chinese preschool children. Contemporary Educational Psychology, 67, 102020. 10.1016/j.cedpsych.2021.102020

[aot70101-bib-0041] * Hui, C. , Snider, L. , & Couture, M. (2016). Self‐regulation workshop and occupational performance coaching with teachers: A pilot study. Canadian Journal of Occupational Therapy, 83(2), 115–125. 10.1177/0008417415627665 27026722

[aot70101-bib-0042] Jeremy, J. , Hinitt, J. , & Spandagou, I. (2025). Interprofessional collaboration: Measuring occupational therapists and teachers' perceptions of collaborative practice in inclusive Australian primary schools. The British Journal of Occupational Therapy, 88(5), 302–313. 10.1177/03080226241295601 40336877 PMC12046194

[aot70101-bib-0043] Jeremy, J. , Spandagou, I. , & Hinitt, J. (2024). Teacher–therapist collaboration in inclusive primary schools: A scoping review. Australian Occupational Therapy Journal, 71(4), 593–611. 10.1111/1440-1630.12931 38320985

[aot70101-bib-0044] Kennedy, S. (2011). Collaboration between occupational therapists and teachers: Definitions, implementation and efficacy. Australian Occupational Therapy Journal, 58(3), 209–214. 10.1111/j.1440-1630.2011.00934.x 21599687

[aot70101-bib-0045] Kennedy, S. , & Stewart, H. (2012). Collaboration with teachers: A survey of south Australian occupational therapists' perceptions and experiences. Australian Occupational Therapy Journal,59(2), 147–155. 10.1111/j.1440-1630.2012.00999.x 22448995

[aot70101-bib-0046] Larsen, L. , Tveit, O. B. , & Holt, T. (2025). Children's school reluctance and satisfaction: The combined role of teacher support and parent–child relation. Scandinavian Journal of Educational Research,70, 1–466. 10.1080/00313831.2025.2492055

[aot70101-bib-0047] Laverdure, P. (2014). Considerations for the development of expert practice in school‐based occupational therapy. Journal of Occupational Therapy, Schools & Early Intervention, 7(3–4), 225–234. 10.1080/19411243.2014.966016

[aot70101-bib-0048] Liew, J. (2012). Effortful control, executive functions, and education: Bringing self‐regulatory and social‐emotional competencies to the table. Child Development Perspectives, 6(2), 105–111. 10.1111/j.1750-8606.2011.00196.x

[aot70101-bib-0049] * Lin, M.‐L. , Paat, Y.‐F. , Cooper, A. , Molina, C. , Smith, E. , Millar, K. , & Fierro, C. (2022). A universal mental health promotion program that demonstrates psychosocial benefits for elementary school students who perceive low emotional self‐efficacy. Journal of Occupational Therapy, Schools & Early Intervention, 16(4), 450–465. 10.1080/19411243.2022.2106342

[aot70101-bib-0050] Lynch, H. , Moore, A. , O'Connor, D. , & Boyle, B. (2023). Evidence for implementing tiered approaches in school‐based occupational therapy in elementary schools: A scoping review. American Journal of Occupational Therapy, 77(1), 7701205110. 10.5014/ajot.2023.050027 36706276

[aot70101-bib-0051] Mac Cobb, S. , Fitzgerald, B. , & Lanigan‐O'Keeffe, C. (2014). The alert program for self‐management of behaviour in second level schools: Results of phase 1 of a pilot study. Emotional and Behavioural Difficulties, 19(4), 410–425. 10.1080/13632752.2014.903593

[aot70101-bib-0052] Martini, R. , Cramm, H. , Egan, M. , & Sikora, L. (2016). Scoping review of self‐regulation: What are occupational therapists talking about? American Journal of Occupational Therapy, 70(6), 1–15. 10.5014/ajot.2016.020362 27767949

[aot70101-bib-0053] Mason, B. K. , Leaf, J. B. , & Gerhardt, P. F. (2023). A research review of the zones of regulation program. Journal of Special Education, 57(4), 219–229. 10.1177/00224669231170202

[aot70101-bib-0054] McClelland, M. M. , & Cameron, C. E. (2019). Developing together: The role of executive function and motor skills in children's early academic lives. Early Childhood Research Quarterly, 46, 142–151. 10.1016/j.ecresq.2018.03.014

[aot70101-bib-0055] McClelland, M. M. , Ponitz, C. C. , Messersmith, E. E. , & Tominey, S. (2010). Self‐regulation. In The handbook of life‐span development. John Wiley & Sons. 10.1002/9780470880166.hlsd001015

[aot70101-bib-0056] McCoy, D. C. (2019). Measuring young children's executive function and self‐regulation in classrooms and other real‐world settings. Clinical Child and Family Psychology Review, 22(1), 63–74. 10.1007/s10567-019-00285-1 30778803

[aot70101-bib-0057] McQuaid, E. (2018). Feasibility study: Implementing the Zones of Regulation® curriculum at a whole‐class level. American Journal of Occupational Therapy, 72(4 Supplement 1), 7211505083p1–7211505083p1. 10.5014/ajot.2018.72S1-PO1014

[aot70101-bib-0058] Miller, A. L. , Lo, S. L. , Bauer, K. W. , & Fredericks, E. M. (2020). Developmentally informed behaviour change techniques to enhance self‐regulation in a health promotion context: A conceptual review. Health Psychology Review, 14(1), 116–131. 10.1080/17437199.2020.1718530 31957556 PMC7254982

[aot70101-bib-0059] Montroy, J. J. , Bowles, R. P. , Skibbe, L. E. , McClelland, M. M. , & Morrison, F. J. (2016). The development of self‐regulation across early childhood. Developmental Psychology, 52(11), 1744–1762. 10.1037/dev0000159 27709999 PMC5123795

[aot70101-bib-0060] Moos, D. C. , & Ringdal, A. (2012). Self‐regulated learning in the classroom: A literature review on the teacher's role. Education Research International, 2012, 1–15. 10.1155/2012/423284

[aot70101-bib-0061] Muir, R. A. , Howard, S. J. , & Kervin, L. (2023). Interventions and approaches targeting early self‐regulation or executive functioning in preschools: A systematic review. Educational Psychology Review, 35(1), 27. 10.1007/s10648-023-09740-6

[aot70101-bib-0062] Novak, I. , & Honan, I. (2019). Effectiveness of paediatric occupational therapy for children with disabilities: A systematic review. Australian Occupational Therapy Journal, 66(3), 258–273. 10.1111/1440-1630.12573 30968419 PMC6850210

[aot70101-bib-0063] Pearson Clinical . (2024). Devereux Behavior Rating Scale – School Form. https://pearsonclinical.in/solutions/devereux-behavior-rating-scale-school-form/

[aot70101-bib-0064] Peters, M. D. J. , Godfrey, C. , McInerney, P. , Khalil, H. , Larsen, P. , Marnie, C. , Pollock, D. , Tricco, A. C. , & Munn, Z. (2022). Best practice guidance and reporting items for the development of scoping review protocols. JBI Evidence Synthesis, 20(4), 953–968. 10.11124/JBIES-21-00242 35102103

[aot70101-bib-0065] Peters, M. D. J. , Marnie, C. , Tricco, A. C. , Pollock, D. , Munn, Z. , Alexander, L. , McInerney, P. , Godfrey, C. M. , & Khalil, H. (2020). Updated methodological guidance for the conduct of scoping reviews. JBI Evidence Synthesis, 18(10), 2119–2126. 10.11124/JBIES-20-00167 33038124

[aot70101-bib-0066] Philpott‐Robinson, K. , Blackwell, D. , Regan, C. , Leonard, C. , Haracz, K. , Lane, A. E. , & Wales, K. (2024). Conflicting definitions of self‐regulation in occupational therapy: A scoping review. Physical & Occupational Therapy in Pediatrics, 45(3), 318–357. 10.1080/01942638.2024.2434468 39632664

[aot70101-bib-0067] Philpott‐Robinson, K. , Johnson, T. , Evans, L. , Wales, K. , Leonard, C. , & Lane, A. E. (2023). Measurement of self‐regulation in preschool and elementary children: A scoping review. Physical & Occupational Therapy in Pediatrics, 43(4), 403–429. 10.1080/01942638.2022.2158055 36647208

[aot70101-bib-0068] Pollock, D. , Peters, M. D. J. , Khalil, H. , McInerney, P. , Alexander, L. , Tricco, A. C. , Evans, C. , de Moraes, E. B. , Godfrey, C. M. , Pieper, D. , Saran, A. , Stern, C. , & Munn, Z. (2023). Recommendations for the extraction, analysis, and presentation of results in scoping reviews. JBI Evidence Synthesis, 21(3), 520–532. 10.11124/JBIES-22-00123 36081365

[aot70101-bib-0069] Portilla, X. , Ballard, P. , Adler, N. , Boyce, W. , & Obradovic, J. (2014). An integrative view of school functioning: Transactions between self‐regulation, school engagement, and teacher‐child relationship quality. Child Development, 85(5), 1915–1931. 10.1111/cdev.12259 24916608 PMC4165700

[aot70101-bib-0070] Rademacher, A. , & Koglin, U. (2019). The concept of self‐regulation and preschoolers' social‐emotional development: A systematic review. Early Child Development and Care, 189(14), 2299–2317. 10.1080/03004430.2018.1450251

[aot70101-bib-0071] Rauvola, R. S. , & Rudolph, C. W. (2023). An operational integration of lifespan development theories. Current Psychology, 42(13), 11184–11194. 10.1007/s12144-021-02385-0

[aot70101-bib-0072] Robson, D. A. , Allen, M. S. , & Howard, S. J. (2020). Self‐regulation in childhood as a predictor of future outcomes: A meta‐analytic review. Psychological Bulletin, 146(4), 324–354. 10.1037/bul0000227 31904248

[aot70101-bib-0073] * Romero‐Ayuso, D. , Espinosa‐Garcia, B. , Gomez‐Marin, E. , Gomez‐Jara, N. , Cuevas‐Delgado, C. , Alvarez‐Benitez, I. , & Trivino‐Juarez, J. M. (2022). A pilot study of improving self‐regulation and social interaction with peers: An “exciting school”. Children (Basel), 9(6), 829. 10.3390/children9060829 35740766 PMC9222160

[aot70101-bib-0074] Rosanbalm, K. , & Murray, D. (2017). Promoting self‐regulation in early childhood: A practice brief. U.S. Department of Health and Human Services. https://eric.ed.gov/?id=ED583624

[aot70101-bib-0075] Seruya, F. M. , & Garfinkel, M. (2020). Caseload and workload: Current trends in school‐based practice across the United States. American Journal of Occupational Therapy, 74(5), 1–8. 10.5014/ajot.2020.039818 32804627

[aot70101-bib-0076] Silkenbeumer, J. , Lüken, L. M. , Holodynski, M. , & Kärtner, J. (2024). Emotion socialization in early childhood education and care—How preschool teachers support children's emotion regulation. Social and Emotional Learning: Research, Practice, and Policy, 4, 100057. 10.1016/j.sel.2024.100057

[aot70101-bib-0077] Smiles, H. , Mechling, C. , Taff, S. D. , & Milton, L. E. (2025). Perspectives of school‐based occupational therapists. The Open Journal of Occupational Therapy, 13(2), 1–10. 10.15453/2168-6408.2233

[aot70101-bib-0078] Smith, M. N. , & Douglas, R. R. (2022). The role of school‐based occupational therapy practitioners in providing tier 1 services to enhance sensory processing and self‐regulation strategies within the classroom. Journal of Occupational Therapy, Schools & Early Intervention, 17(1), 26–36. 10.1080/19411243.2022.2129904

[aot70101-bib-0079] Solomon, T. , Plamondon, A. , O'Hara, A. , Finch, H. , Goco, G. , Chaban, P. , Huggins, L. , Ferguson, B. , & Tannock, R. (2018). A cluster randomized‐controlled trial of the impact of the tools of the mind curriculum on self‐regulation in Canadian preschoolers. Frontiers in Psychology, 8, 2366. 10.3389/fpsyg.2017.02366 29403411 PMC5782823

[aot70101-bib-0080] * Teselle, A. (2021). Linking body cues to emotions for elementary aged children: An understanding by design curriculum for social‐emotional learning (Publication No. 28497854) [Doctoral dissertation, Boston University]. ProQuest Dissertations & Theses. https://www.proquest.com/dissertations-theses/linking-body-cues-emotions-elementary-aged/docview/2536767440/se-2

[aot70101-bib-0081] The Canadian Occupational Performance Measure . (2024). The COPM is an individualized, client‐centred outcome measure. https://www.thecopm.ca/

[aot70101-bib-0082] TherapyWorks . (2023). Alert Program® overview: Supporting children with autism. https://www.alertprogram.com/alert-program-overview-supporting-children-with-autism/#:~:text=The%20Alert%20Program%C2%AE%20is%20based%20on%20how%20the%20body,well%20as%20movement%20and%20gravity

[aot70101-bib-0083] Timmons, K. , Pelletier, J. , & Corter, C. (2016). Understanding children's self‐regulation within different classroom contexts. Early Child Development and Care, 186(2), 249–267. 10.1080/03004430.2015.1027699

[aot70101-bib-0084] Torrington, J. , Bower, M. , & Burns, E. C. (2023). Elementary students' self‐regulation in computer‐based learning environments: How do self‐report measures, observations and teacher rating relate to task performance? British Journal of Educational Technology, 55, 231–258. 10.1111/bjet.13338

[aot70101-bib-0085] Tricco, A. C. , Lillie, E. , Zarin, W. , O'Brien, K. K. , Colquhoun, H. , Levac, D. , Moher, D. , Peters, M. D. J. , Horsley, T. , Weeks, L. , Hempel, S. , Akl, E. A. , Chang, C. , McGowan, J. , Stewart, L. , Hartling, L. , Aldcroft, A. , Wilson, M. G. , Garritty, C. , … Straus, S. E. (2018). PRISMA extension for scoping reviews (PRISMA‐ScR): Checklist and explanation. Annals of Internal Medicine, 169(7), 467–473. 10.7326/m18-0850 30178033

[aot70101-bib-0086] * Van Waelvelde, H. , De Roubaix, A. , Steppe, L. , Troubleyn, E. , De Mey, B. , Dewitte, G. , Debrabant, J. , & Van de Velde, D. (2017). Effectiveness of a self‐regulated remedial program for handwriting difficulties. Scandinavian Journal of Occupational Therapy, 24(5), 311–319. 10.1080/11038128.2017.1282041 28276960

[aot70101-bib-0087] Varghese, C. , Vernon‐Feagans, L. , & Bratsch‐Hines, M. (2019). Associations between teacher–child relationships, children's literacy achievement, and social competencies for struggling and non‐struggling readers in early elementary school. Early Childhood Research Quarterly, 47, 124–133. 10.1016/j.ecresq.2018.09.005

[aot70101-bib-0088] Veritas Health Innovation . (2024). Covidence systematic review software. www.covidence.org

[aot70101-bib-0089] Vincent, R. , Stewart, H. , & Harrison, J. (2008). South Australian school teachers' perceptions of occupational therapy reports. Australian Occupational Therapy Journal, 55(3), 163–171. 10.1111/j.1440-1630.2007.00703.x 20887458

[aot70101-bib-0090] Vink, M. , Gladwin, T. E. , Geeraerts, S. , Pas, P. , Bos, D. , Hofstee, M. , Durston, S. , & Vollebergh, W. (2020). Towards an integrated account of the development of self‐regulation from a neurocognitive perspective: A framework for current and future longitudinal multi‐modal investigations. Developmental Cognitive Neuroscience, 45, 100829. 10.1016/j.dcn.2020.100829 32738778 PMC7394770

[aot70101-bib-0091] * Wagner, B. , Latimer, J. , Adams, E. , Carmichael Olson, H. , Symons, M. , Mazzucchelli, T. , Jirikowic, T. , Watkins, R. , Cross, D. , Carapetis, J. , Boulton, J. , Wright, E. , McRae, T. , Carter, M. , & Fitzpatrick, J. (2020). School‐based intervention to address self‐regulation and executive functioning in children attending primary schools in remote Australian Aboriginal communities. PLoS ONE, 15(6), e0234895. 10.1371/journal.pone.0234895 32579567 PMC7314028

[aot70101-bib-0092] Williams, K. E. , Bentley, L. A. , Savage, S. , Eager, R. , & Nielson, C. (2023). Rhythm and movement delivered by teachers supports self‐regulation skills of preschool‐aged children in disadvantaged communities: A clustered RCT. Early Childhood Research Quarterly, 65, 115–128. 10.1016/j.ecresq.2023.05.008

[aot70101-bib-0093] World Health Organization . 2002. ICF Beginner's Guide: Towards a Common Language for Functioning, Disability and Health. https://www.who.int/publications/m/item/icf-beginner-s-guide-towards-a-common-language-for-functioning-disability-and-health

[aot70101-bib-0094] Wyatt, T. M. , Denham, S. A. , & Bassett, H. H. (2025). Self‐regulation in preschool children: Hot and cool executive control as predictors of later classroom learning behaviors. Learning and Individual Differences, 121, 102701. 10.1016/j.lindif.2025.102701 40584516 PMC12199747

[aot70101-bib-0095] Zakszeski, B. , Hojnoski, R. L. , Dever, B. V. , DuPaul, G. J. , & McClelland, M. M. (2020). Early elementary trajectories of classroom behavior self‐regulation: Prediction by student characteristics and malleable contextual factors. School Psychology Review, 49(2), 161–177. 10.1080/2372966X.2020.1717373

