# Supplementary material for: Occupational therapists, self‐regulation, and primary schools: A scoping review
Source: Aust Occup Ther J. 2026 Jul 12;73(4):e70101. doi: 10.1111/1440-1630.70101 (PMC13357039; doi:10.1111/1440-1630.70101)
Supplement: Supplementary file 1 — Data S1. Supporting Information. [file AOT-73-0-s001.docx]

Supplementary Material - Full search strategy for CINAHL Complete

((mapped heading "Self Regulation") OR ("self regulat*" or "selfregulat*" or "self control*" or "selfcontrol*")) AND ((mapped heading "Occupational Therapy") OR ("occupational therap*")) AND (((mapped heading "Schools, Elementary") OR (mapped heading "Schools, Middle") OR (mapped heading "Schools, Nursery") OR (mapped heading "Schools, Secondary") OR (mapped heading "Schools, Special")) OR ("education*" or "classroom*" or "class room*") OR ("primary school*" or "high school*" or "preschool*" or "pre school*" or "kindergarten*")).
